# A functional glycogen biosynthesis pathway in *Lactobacillus acidophilus*: expression and analysis of the *glg* operon

**DOI:** 10.1111/mmi.12338

**Published:** 2013-08-16

**Authors:** Yong Jun Goh, Todd R Klaenhammer

**Affiliations:** Department of Food, Bioprocessing and Nutrition Sciences, North Carolina State UniversityRaleigh, NC, 27695, USA

## Abstract

Glycogen metabolism contributes to energy storage and various physiological functions in some prokaryotes, including colonization persistence. A role for glycogen metabolism is proposed on the survival and fitness of *Lactobacillus acidophilus*, a probiotic microbe, in the human gastrointestinal environment. *L. acidophilus* NCFM possesses a glycogen metabolism (*glg*) operon consisting of *glgBCDAP-amy-pgm* genes. Expression of the *glg* operon and glycogen accumulation were carbon source- and growth phase-dependent, and were repressed by glucose. The highest intracellular glycogen content was observed in early log-phase cells grown on trehalose, which was followed by a drastic decrease of glycogen content prior to entering stationary phase. In raffinose-grown cells, however, glycogen accumulation gradually declined following early log phase and was maintained at stable levels throughout stationary phase. Raffinose also induced an overall higher temporal *glg* expression throughout growth compared with trehalose. Isogenic Δ*glgA* (glycogen synthase) and Δ*glgB* (glycogen-branching enzyme) mutants are glycogen-deficient and exhibited growth defects on raffinose. The latter observation suggests a reciprocal relationship between glycogen synthesis and raffinose metabolism. Deletion of *glgB* or *glgP* (glycogen phosphorylase) resulted in defective growth and increased bile sensitivity. The data indicate that glycogen metabolism is involved in growth maintenance, bile tolerance and complex carbohydrate utilization in *L. acidophilus*.

## Introduction

*Lactobacillus acidophilus* is one of the most recognized and widely used probiotic microbes in the commercial production of yogurt and dietary supplements (Sanders and Klaenhammer, 2001). It belongs to a phylogenetically diverse genus that includes species found naturally in milk, plants, meats and the mucosal surfaces [oral, gastrointestinal (GI) tract and reproductive tracts] of mammalian hosts, as well as species that were domesticated for centuries, which altogether represent a group of microorganisms that are of economic importance for the manufacturing of fermented foods and as probiotic adjuncts in cultured dairy products. The probiotic functionality of *L. acidophilus* has been well documented, including the alleviation of lactose intolerance (Kim and Gilliland, 1983), mitigation of cold and influenza-like symptoms in children (Leyer *et al.*, 2009), the modulation of immune cell functions (Konstantinov *et al.*, 2008) and the alleviation of abdominal pain via modulation of the visceral pain perception (Rousseaux *et al.*, 2007). A *L. acidophilus* NCFM derivative deficient in cell wall lipoteichoic acid also showed potential for the treatment of colitis and colonic polyposis (Mohamadzadeh *et al.*, 2011; Khazaie *et al.*, 2012). In addition, due to its Generally Regarded As Safe (GRAS) status and the ability to survive transit through the GI tract, *L. acidophilus* has been considered as an ideal vehicle for mucosal-targeted delivery of vaccines and biotherapeutics (Mohamadzadeh *et al.*, 2009; Kajikawa *et al.*, 2012). Consequently, extensive studies are ongoing to elucidate *in vivo* mechanisms involved in the stress adaptation and host–microbe interactions of *L. acidophilus* in order to enhance its biodelivery and fitness in the host GI environment.

Glycogen synthesis in bacteria is commonly regarded as a mechanism to prolong survival by supplying the energy for maintenance, thereby allowing cells to sense and respond to changing environments, such as starvation and stress (Preiss, 1984). Structurally analogous to amylopectin, an energy storage component of starch in plants, glycogen is a soluble α-1,4-linked glucose polymer with comparatively more extensive α-1,6-linked branches. Glycogen is considered a flexible and efficient form of a reserve compound due to its large molecular mass, highly branched structure and its accumulation poses little effect on the internal osmotic pressure in the cells. In most bacteria, glycogen biosynthesis generally occurs in the presence of excess carbon sources under limited growth conditions (Preiss, 1984). Glycogen synthesis in bacteria involves glucose-1-phosphate adenylyltransferase (GlgC or GlgCD), glycogen synthase (GlgA) and glycogen branching enzyme (GlgB). Glucose-1-phosphate serves as a substrate for ADP-glucose synthesis catalysed by GlgC or GlgCD. Then, GlgA catalyses the transfer of glucosyl units from ADP-glucose to the elongating chain of linear α-1,4-glucan, where GlgB subsequently cleaves off a portion of the glucan and attaches it to existing chains via α-1,6 linkages to form the glycogen structure. Glycogen catabolism involves glycogen phosphorylase (GlgP) (Alonso-Casajus *et al.*, 2006) and glycogen-debranching enzyme (GlgX) (Dauvillee *et al.*, 2005; Seibold and Eikmanns, 2007), which catalyses the gradual breakdown of the glucan chain of glycogen and debranching of the phosphorylase-limit dextrins respectively.

An increasing number of studies have revealed the involvement of glycogen metabolism in a multitude of physiological functions, such as sporulation in *Bacillus subtilis* (Kiel *et al.*, 1994) and the biosynthesis of trehalose in *Corynebacterium glutamicum* which contributes to osmoprotection and cell wall synthesis (Tzvetkov *et al.*, 2003; Seibold and Eikmanns, 2007). In *Mycobacterium smegmatis* and several other microorganisms, glycogen synthesis and degradation occurred in parallel during early growth phase (Belanger and Hatfull, 1999; Dauvillee *et al.*, 2005; Seibold and Eikmanns, 2007). This led to a proposed model that glycogen metabolism functions as a carbon capacitor that regulates the downstream carbon and energy fluxes (Belanger and Hatfull, 1999). Similarly, in *Escherichia coli*, glycogen metabolism is integral in the complex network of major cellular processes including energy production, carbohydrate and amino acid metabolism, stress responses and cell–cell communication, and is tightly regulated by nutritional and energy status (Eydallin *et al.*, 2007; 2010). The ability to synthesize and accumulate glycogen has also been associated with the colonization persistence of *Streptococcus mutans* (Busuioc *et al.*, 2009), *Mycobacterium tuberculosis* (Sambou *et al.*, 2008) and *E. coli* (Jones *et al.*, 2008), indicating glycogen synthesis as an important niche factor in the host environments.

Overall, these studies suggest that glycogen metabolism potentially contributes to the probiotic activities and retention of *L. acidophilus* in the GI environment. The presence of a putative glycogen metabolism gene cluster in the genome of *L. acidophilus* NCFM (Altermann *et al.*, 2005) and certain lactobacilli species mostly associated with natural or mammalian host environments, prompted us to explore the role of glycogen metabolism in the physiological functions and host fitness of probiotic microbes. In this work, we present the first example of a functional glycogen synthesis pathway in probiotic lactic acid bacteria (LAB). Glycogen metabolism in *L. acidophilus* NCFM is dependent on the type of carbon sources and growth phases. Inactivation of the glycogen metabolism pathway genes revealed their involvement in growth, bile tolerance and potentially the metabolism of complex carbohydrates.

## Results

### Identification and comparative sequence analysis of the putative glycogen operon in *L. acidophilus*

**Fig 1:**
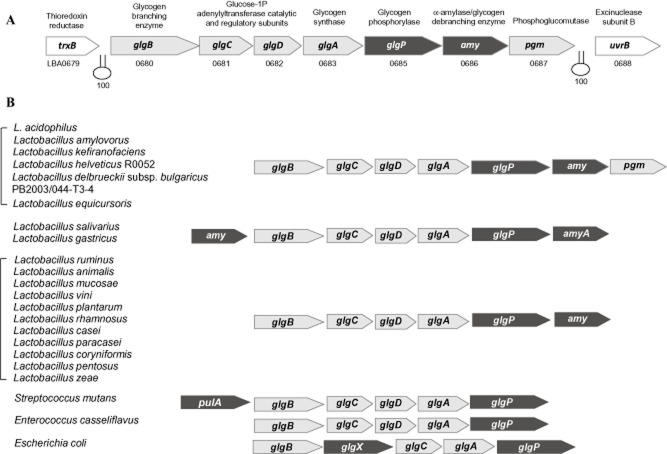
A. Organization of the glycogen metabolism gene cluster (*glg-amy-pgm*) encoded in the *L. acidophilus* NCFM genome. Predicted rho-independent transcriptional terminators are indicated in hairpin loops with overall confidence score (ranges from 0 to 100).B. Comparative structural organization of glycogen metabolism gene clusters in other microorganisms. The gene cluster is present in a human microbiome reference strain of *L. bulgaricus* (strain PB2003/044-T3-4) and a probiotic strain of *L. helveticus* (strain R0052) but not in other sequenced dairy-associated *L. bulgaricus* and *L. helveticus* strains. Putative glycogen synthesis genes are indicated in light grey arrows, whereas glycogen degradation genes are indicated in black arrows. *amyA* or *pulA*, α-amylase/pullulanase/glycogen-debranching enzyme, *glgX*, glycogen-debranching enzyme.

The putative glycogen metabolic pathway in *L. acidophilus* is encoded by an 11.7 kb gene cluster consisting of seven genes: *glgB* (LBA0680; putative glycogen-branching enzyme), *glgC* and *glgD* (LBA0681 and LBA0682; putative glucose-1-phosphate adenylyltransferase catalytic and regulatory subunits respectively), *glgA* (LBA0683; putative glycogen synthase), *glgP* (LBA0685; putative glycogen phosphorylase), *amy* (LBA0686; putative α-amylase) and *pgm* (LBA0687; putative phosphoglucomutase) (Fig. 1A). The glycogen gene cluster is flanked by a thioredoxin reductase gene (LBA0679; *trxB*) and UvrBA excinuclease genes (LBA0688 and LBA0689; *uvrBA*) on the same orientation. Rho-independent transcriptional terminators were predicted downstream of *trxB* and *pgm* (Kingsford *et al.*, 2007), suggesting that the *glgBCDAP-amy-pgm* gene cluster constitutes a polycistronic operon.

To identify potential glycogen pathways in other *Lactobacillus* species, a blast search was performed based on the putative glycogen metabolic enzymes of *L. acidophilus* NCFM. From the genome sequences of 59 *Lactobacillus* species available to date in the NCBI genome sequence database, only 19 species of *Lactobacillus* harboured a similar set of glycogen metabolic genes. Most of these species are associated with mammalian hosts or natural environments. Interestingly, the gene cluster is absent in closely related dairy-domesticated strains of *Lactobacillus helveticus* and *Lactobacillus delbrueckii* subsp. *bulgaricus*, but was detected in non-dairy originated strains of *L. helveticus* (strain R0052, a commercial probiotic strain isolated from sweet acidophilus milk) (Tompkins *et al.*, 2012) and *L. bulgaricus* (Human Microbiome Project reference strain PB2003/044-T3-4; isolated from the human vaginal cavity), indicating a niche-specific function of glycogen metabolism among lactobacilli.

**Fig 2:**
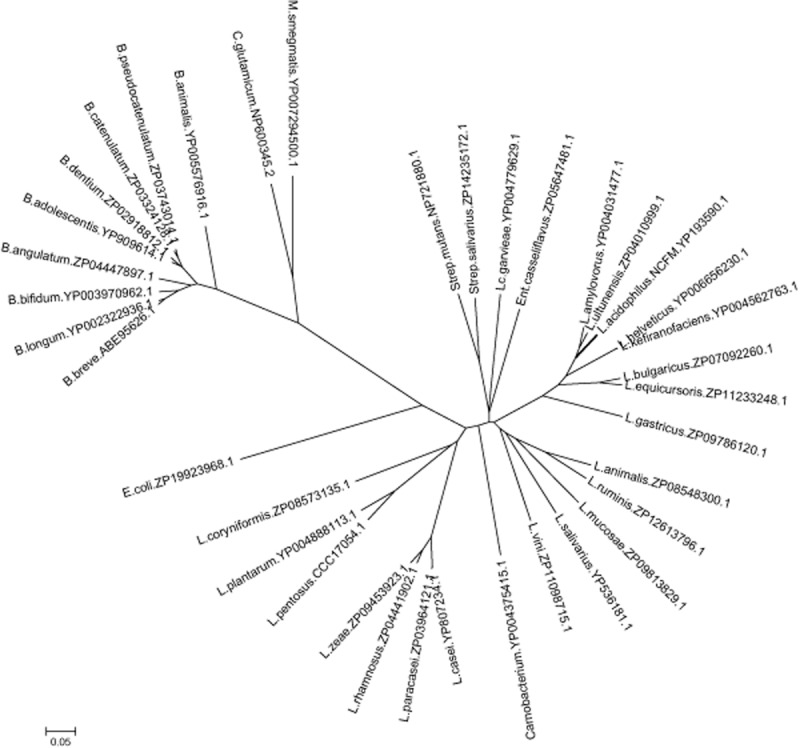
Phylogenetic tree of GlgA orthologues among lactobacilli and other microorganisms. The protein sequences were aligned and the phylogenetic tree was constructed using clustalx v2.1 and visualized using MEGA 5.1 software. The species names followed by the GenBank accession number of the corresponding GlgA proteins are indicated. Abbreviated genus: *B.*, *Bifidobacterium*; *C.*, *Corynebacterium*; *E.*, *Escherichia*; *Ent*., *Enterococcus*; *L.*, *Lactobacillus*; *Lc.*, *Lactococcus*; *M.*, *Mycobacterium*; *Strep*., *Streptococcus*. The scale bar indicates an evolutionary distance of 0.05 amino acid substitutions per position.

BlastP analysis of the deduced amino acid sequences of the glycogen pathway enzymes from *L. acidophilus* NCFM revealed sequence homology ranging from 41% to 90% to the corresponding proteins among the *glg*-encoding lactobacilli, with closest orthologues found in strains of *Lactobacillus ultunensis*, *Lactobacillus amylovorus*, *L. helveticus* R0052, *L. bulgaricus* PB2003/044-T3-4, *Lactobacillus kefiranofaciens* and *Lactobacillus equicursoris*. A phylogenetic tree of putative glycogen synthases, GlgA, a conserved glycogen synthesis enzyme, was constructed to provide an overview of the relative phylogenetic relationship among the glycogen gene clusters encoded in lactobacilli. Figure 2 showed that the GlgA from lactobacilli are clustered into three major distinct groups that resembled the phylogenetic relationship based on 16S rRNA gene sequences (Felis *et al.*, 2009). This observation further implies that the presence of this gene cluster among specific *Lactobacillus* species was not a result of recent horizontal gene acquisition. Interestingly, the putative GlgA from strains of the *Lactobacillus rhamnosus*–*L. casei* group and *Lactobacillus plantarum*–*L. pentosus* group, which exhibited the least sequence homology (∼ 50% identity) with the GlgA of *L. acidophilus*, are clustered between the corresponding orthologues from *E. coli* and *Carnobacterium* species, indicating a major divergence of the enzymes from their *Lactobacillus* counterparts.

Genome sequence mining showed that most of the compared species have a conserved chromosomal mosaic arrangement of the *glg* genes, with the *glgBCDAP* genes representing the core genes in these species (Fig. 1B). Characteristically, all *Lactobacillus* species possess a copy of an *amy* gene, and only species closely related to *L. acidophilus* possess a *pgm* gene downstream of the core genes. These latter species were also grouped within the same phylogenetic cluster based on their GlgA sequences (Fig. 2), supporting the phylogenetic prediction of the *glg* gene sets based on the sequence conservation of GlgA. The conserved gene regions flanking the *glg* locus in *L. acidophilus*, *L. ultunensis*, *L. amylovorus*, *L. helveticus* and *L. kefiranofaciens* further suggest that these species shared a common ancestor. Reverse-transcriptase (RT)-PCR assay confirmed that the *glg-amy-pgm* genes of *L. acidophilus* NCFM are co-transcribed as an operon (Fig. S1), which may also be the case for other *Lactobacillus* species with similar glycogen metabolism gene sets. *Bifidobacterium* species that are known as GI commensals and probiotic microorganisms were also included in the analysis. The glycogen pathway genes in *Bifidobacterium* species, like in the other species of Actinobacteria, *C. glutamicum* (Seibold *et al.*, 2007), are not organized as an operon-like structure, but rather scattered within the genomes. This distinct difference also reflects the distant phylogenetic relatedness of its GlgA proteins with the other species.

### Expression of the *glg* operon and glycogen accumulation is carbon source- and growth phase-dependent

#### Gene expression and glycogen accumulation on various carbohydrate sources

**Fig 3:**
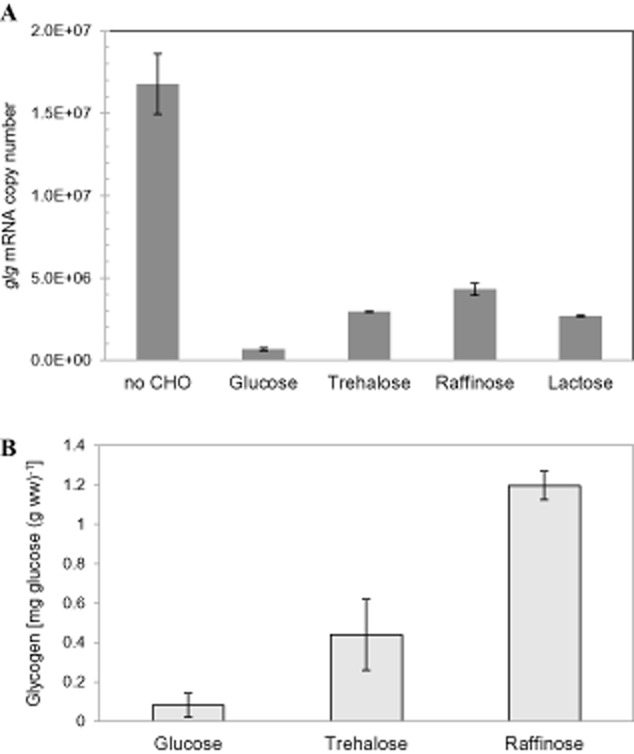
Transcript levels of the *glg* operon (A) and intracellular glycogen content (B) in *L. acidophilus* grown in SDM supplemented with 2% (w/v) of various carbohydrates or no carbohydrate (no CHO). Intracellular glycogen content was expressed as mg of glucose (released from glycogen by amyloglucosidase) per g of cell wet weight. The data are the mean ± standard deviation for two independent biological replicates.

Previous transcriptomic analysis of carbohydrate catabolism by *L. acidophilus* revealed that the *glg* operon was expressed at higher levels when raffinose or trehalose was provided as a sole carbon source compared with other sugars; whereas glucose appeared to repress the *glg* genes (Barrangou *et al.*, 2006). In the present study, the expression of the *glg* operon at mid-log phase (OD_600_ ∼ 0.6) during growth in semi-defined medium (SDM) containing 2% raffinose, trehalose, lactose, glucose, or without carbohydrate supplementation (control), was examined by real time-quantitative PCR (RT-qPCR) experiments with primers targeting the *glgA* gene of the polycistronic cDNA. Comparison of the transcript levels confirmed that growth on raffinose resulted in a slightly higher expression than on trehalose or lactose, which overall were at four- to sixfold higher transcription level of *glgA* as compared with its transcription when cells were grown on glucose (Fig. 3A). Accordingly, the glycogen content in cells grown on 2% raffinose, trehalose or glucose showed a similar trend where the highest and lowest levels of intracellular glycogen were recovered from raffinose- [1.20 ± 0.07 mg glucose (g ww)^−1^] and glucose-grown cells [0.08 ± 0.06 mg glucose (g ww)^−1^] respectively (Fig. 3B). The results from glucose-grown cells indicate that glycogen metabolism is likely subjected to carbon catabolite regulation, which is supported by the previous prediction of a catabolite responsive element (*cre*) sequence (AGTAAGCGCTTTCC) upstream of the *glg* operon (Barrangou *et al.*, 2006). Unexpectedly, the transcript level of the *glg* operon was significantly elevated in medium without carbohydrate supplementation, which was approximately 25-fold higher when compared with cells grown on glucose.

#### Comparison of temporal glycogen accumulation profiles and *glg* expression during growth on raffinose and trehalose

**Fig 4:**
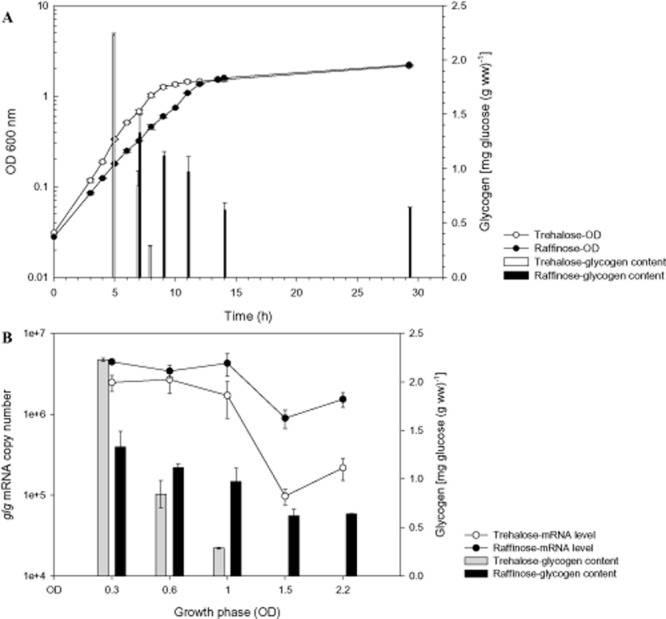
A. Growth and glycogen accumulation profiles of *L. acidophilus* in SDM containing 2% trehalose or raffinose. The intracellular glycogen contents under both carbohydrate conditions at various growth phases (indicated by OD_600_) from (A) were compiled in (B) and plotted against the transcript levels of the *glg* operon. The data represent the mean ± standard deviation for two independent biological replicates.

Since the *glg* operon was inducible by raffinose and trehalose, we further examined the intracellular glycogen profiles as well as the *glg* expression at various growth phases in SDM with 2% raffinose or trehalose (Fig. 4A). A lower growth rate was observed for the raffinose-grown cells [specific growth rate (μ) = 0.31 h^−1^ compared with the trehalose-grown cells, μ = 0.42 h^−1^], although the culture eventually reached a final cell density that is equivalent to that of the trehalose-grown cells (OD_600_ 2.2). Both cultures displayed a dramatic difference in their temporal glycogen accumulation profiles. On both sugars, the highest glycogen content was observed during early log phase, indicating that *L. acidophilus* was actively accumulating glycogen during the early growth phase (Fig. 4A). Similar observations have been made in *C. glutamicum*, where glycogen accumulated rapidly at early log phase when growing on various sugar substrates (Seibold *et al.*, 2007). In *L. acidophilus* cells grown on trehalose, a drastic decrease of glycogen content occurred following early log phase, and eventually dropped to undetectable levels when the cells entered stationary stage. In the case of raffinose-grown cells, the glycogen level gradually declined and dropped by approximately 50% as the cells entered stationary phase, presumably due to the decrease of raffinose availability as a carbon source for glycogen synthesis. Remarkably, the intracellular glycogen content of the cells remained at a stable level even at late stationary phase.

RT-qPCR analysis revealed maximal *glg* expression levels from early log through early stationary phase for both raffinose- and trehalose-grown cells (Fig. 4B). This is followed by a decrease in transcript levels during stationary phase, and surprisingly, a gradual secondary induction of the *glg* genes as cells approaching late stationary phase. This secondary induction may correspond to the upregulation of the *glg* operon observed previously in cells grown in SDM without added carbon source (Fig. 3A). Overall, the genes were consistently expressed at a higher level on raffinose throughout the growth phase (Fig. 4B). This is particularly evident during the later growth phases, where *glg* was transcribed at approximately nine- and sevenfold higher on raffinose than on trehalose during stationary and late stationary phases respectively. This result was further reflected by the undetectable glycogen level in the trehalose-grown cells during these stationary-phase periods. It is interesting to note that, despite a significantly higher glycogen content in the trehalose-grown cells during early log phase, the expression of the operon was slightly lower compared with that of raffinose-grown cells. Also, transcription of the operon remained at high levels on trehalose during the early growth phases although the glycogen levels decreased sharply. Since the glycogen synthesis and degradation genes are co-transcribed, it is likely that the decrease in glycogen level at mid-log phase was a result of post-transcriptional regulation that led to a shift of increased glycogen-degrading activity or a decrease of glycogen synthesis. Collectively, the temporal expression trend of the *glg* operon did not show a strong correlation to the glycogen accumulation status of the cells, especially on trehalose.

### Phenotypic analyses of glycogen metabolism mutants

In order to investigate the proposed functions of the *glg* operon in glycogen metabolism and its potential role in probiotic-relevant phenotypes, four genes, two of which are predicted to be involved in glycogen anabolism (*glgA* and *glgB*) and catabolism (*glgP* and *amy*), were targeted for chromosomal inactivation. In-frame deletions of *glgA*, *glgB*, *glgP* or *amy* gene were constructed in the *L. acidophilus* Δ*upp* background host (NCK1909) using a *upp*-based counterselective gene replacement system (Goh *et al.*, 2009). A panel of isogenic mutants were generated and designated as Δ*glgA*, Δ*glgB*, Δ*glgP* and Δ*amy* (NCK2180, NCK2277, NCK2182 and NCK2280 respectively; Table 1) where ≥ 93% of each of the respective genes were deleted from the chromosome.

**Table 1:** Bacterial strains and plasmids used in this study

	Genotype or characteristics	Source or reference
Strains		
*L. acidophilus*		
NCFM	Human intestinal isolate	Barefoot and Klaenhammer (1983)
NCK1909	NCFM carrying a 315 bp in-frame deletion within the *upp* gene; parent/background strain for all deletion mutants	Goh *et al.* (2009)
NCK1910	NCK1909 harbouring pTRK669; host for pORI-based counterselective integration vector	Goh *et al.* (2009)
NCK2180	NCK1909 carrying a 1347 bp in-frame deletion within *glgA* (LBA0683)	This study
NCK2277	NCK1909 carrying a 1779 bp in-frame deletion within *glgB* (LBA0680)	This study
NCK2182	NCK1909 carrying a 2280 bp in-frame deletion within *glgP* (LBA0685)	This study
NCK2280	NCK1909 carrying a 1674 bp in-frame deletion within *amy* (LBA0686)	This study
*E. coli*		
EC101	RepA^+^ JM101; kanamycin-resistant; *repA* from pWV01 integrated in chromosome; cloning host for pORI-based plasmids	Law *et al.* (1995)
Plasmids		
pTRK935	3.0 kb; pORI-based counterselective integration vector containing a native *L. acidophilus* NCFM *upp* expression cassette and *lacZ'* multiple cloning sites from pUC19	Goh *et al.* (2009)
pTRK1042	4.6 kb; pTRK935 with a mutated copy of *glgA* cloned into BamHI/SacI sites	This study
pTRK1071	4.3 kb; pTRK935 with a mutated copy of *glgB* cloned into HindIII/EcoRI sites	This study
pTRK1043	4.3 kb; pTRK935 with a mutated copy of *glgP* cloned into BamHI/SacI sites	This study
pTRK1072	4.3 kb; pTRK935 with a mutated copy of *amy* cloned into BamHI/SacI sites	This study

#### Glycogen biosynthesis requires functional *glgA* and *glgB*

**Fig 5:**
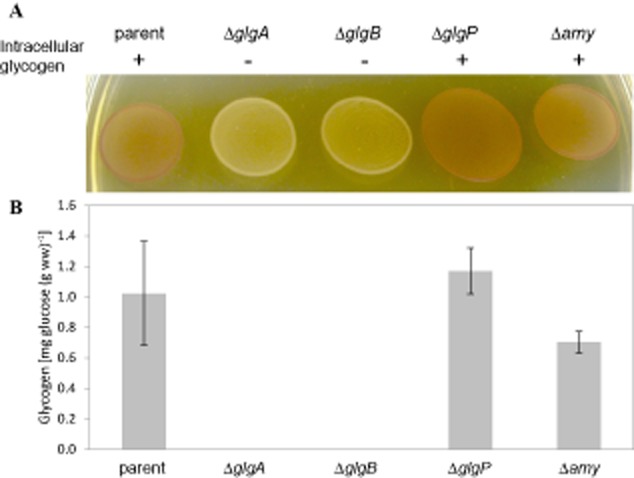
A. Iodine staining of *L. acidophilus* NCK1909 parent strain and glycogen metabolism mutant colonies grown on solid SDM medium containing 2% trehalose. Both Δ*glgA* and Δ*glgB* mutant cells appeared as yellow/colourless indicative of glycogen-deficient phenotype. Like the parent cells, the Δ*glgP* and Δ*amy* mutants were stained brown, indicating that their ability to synthesize intracellular glycogen was unaffected.B. Intracellular glycogen content of mid-log-phase cells cultivated in SDM containing 2% trehalose. Glycogen was not detected from both Δ*glgA* and Δ*glgB* cultures. Data shown represent the mean ± standard deviation for two independent biological replicates.

The ability of the various mutants to synthesize glycogen was qualitatively determined by an iodine staining method. Figure 5A showed that the Δ*glgP* and Δ*amy* mutants were stained brown from the formation of I_2_-polysaccharide complex, implying the presence of intracellular glycogen at levels at least comparable to the parent strain (NCK1909), and that their ability to synthesize glycogen was unaffected. On the other hand, both the Δ*glgA* and Δ*glgB* mutant cells appeared as yellow to colourless, indicating a glycogen-deficient phenotype. To further confirm the glycogen phenotype of the mutants by quantitative glycogen measurement assay, all strains were grown in SDM with 2% trehalose and harvested at mid-log phase. Results as depicted in Fig. 5B verified the absence of intracellular glycogen in both the Δ*glgA* and the Δ*glgB* mutants. Therefore, the inactivation of *glgA* or *glgB* alone abolished the ability of the mutants to synthesize glycogen, confirming that (i) the *glg* operon is functional in *L. acidophilus*, (ii) *glgA* and *glgB* encodes for a glycogen synthase and a glycogen-branching enzyme, respectively, and (iii) both functional *glgA* and *glgB* are required for the formation of glycogen by *L. acidophilus*.

#### Inactivation of *glgB* or *glgP* affects growth

When the growth of the mutant strains was monitored in MRS broth, both Δ*glgB* and Δ*glgP* showed slightly slower growth compared with the parent strain (μ = 0.38 h^−1^, 0.37 h^−1^ and 0.48 h^−1^ respectively) (Fig. S2). Eventually both mutant strains reached similar cell counts to the parent during stationary phase (at 12.5 h and 14 h), although their optical densities remained slightly lower. When cultured on solid MRS medium, the Δ*glgB* and Δ*glgP* cells appeared as a mixed population of large and small colonies, and required at least an additional 24 h incubation period to reach a comparable colony size to the parent. Microscopic examination of both cultures, however, did not show any significant difference in the cellular morphology compared with the parent strain. To eliminate the possibility of culture contamination, the purity of the mutated genotypes of these mixed morphotype populations was verified by PCR assay. Thus, aside from slower growth, the morphotype variant of the Δ*glgB* and Δ*glgP* cells could partly result in the slightly lower optical density values of the broth cultures during stationary phase, despite their comparable cell counts (cfu ml^−1^) relative to the parent strain.

#### Glycogen synthesis pathway is required for efficient growth on raffinose

**Fig 6:**
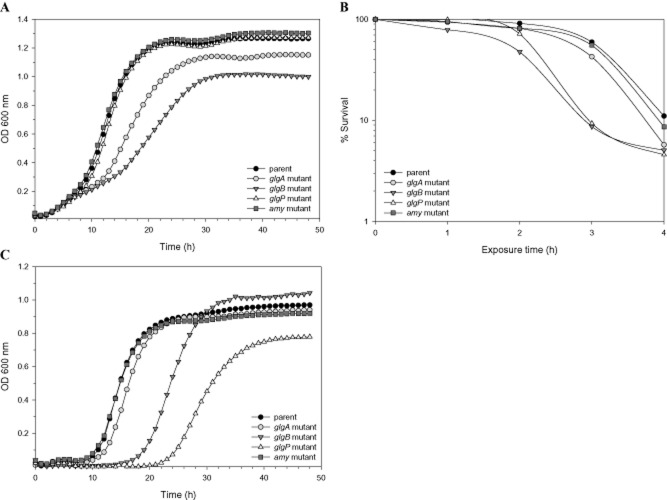
A. Growth of *L. acidophilus* glycogen metabolism mutants compared with the parent strain in SDM containing 1% raffinose.B. Survival of the strains in simulated small intestinal juice (SIJ; contains 0.3% Oxgall bile). Percentage of survival represents viable cells (cfu ml^−1^) at various time points (1, 2, 3 and 4 h) versus at time zero.C. Growth in MRS broth supplemented with 0.5% Oxgall bile. The data shown are representation of at least three independent biological replicates.

Since the presence of raffinose appeared to upregulate the expression of the *glg* operon and glycogen accumulation in *L. acidophilus*, we sought to determine whether the mutation of the *glg* genes would affect growth on raffinose as a sole carbon source. Each mutant strain, previously grown in MRS (typically contains 2% glucose), was used to inoculate (1% inoculum) into SDM supplemented with 1% raffinose, and growth was monitored by OD_600_ measurement. Interestingly, both Δ*glgA* and Δ*glgB* mutants exhibited weaker growth in the raffinose medium compared with the parent strain (μ = 0.13 h^−1^, 0.08 h^−1^ and 0.19 h^−1^ respectively), whereas the growth of the other mutants was unaffected (Fig. 6A). Meanwhile, all mutant strains showed normal growth on trehalose (data not shown). Subsequently, to confirm that the observed growth phenotypes of Δ*glgA* and Δ*glgB* mutants on raffinose was not affected by the pre-exposure to the repression effect of glucose, both the Δ*glgA* mutant and the parent strain were transferred once in SDM with 1% of either trehalose (as an inducer of the *glg* operon) or glucose (control), and the respective overnight cultures were used for growth experiments on raffinose. Regardless of the pre-exposed carbohydrate source, the *glgA* mutant consistently displayed a growth defect phenotype with raffinose as a carbon source (data not shown). These results indicate an important role of *glgA* and *glgB* genes, and presumably glycogen synthesis, in raffinose metabolism by *L. acidophilus*.

#### Sensitivity of Δ*glgB* and Δ*glgP* mutants to simulated small intestinal juice and bile

To determine whether glycogen metabolism plays a role in tolerance to GI transit, the mutant strains were subjected to challenge assays in simulated gastric juice (SGJ) and small intestine juice (SIJ). All *glg* mutants demonstrated a survival rate in SGJ that was comparable to the parent strain (data not shown), indicating a less direct role of the *glg* genes in acid tolerance. On the other hand, after a 3 h exposure period in SIJ, the percentage of survivors for both Δ*glgB* and Δ*glgP* mutants were decreased to 9% compared with that of 60%, 55% and 43% for the parent, Δ*amy* and Δ*glgA* mutants respectively (Fig. 6B). Aside from the lower survival rate, the recovered colonies of Δ*glgB* and Δ*glgP* also appeared much smaller than the other strains after 48 h of incubation. A distinctly longer lag phase was observed for the Δ*glgB* (μ = 0.23 h^−1^) and Δ*glgP* (μ = 0.22 h^−1^) mutants compared with the parent (μ = 0.33 h^−1^) and other strains when grown in the presence of 0.5% (w/v) Oxgall bile (Fig. 6C). This indicates that the SIJ sensitivity phenotype of the two mutants is likely attributed to their decreased tolerance to bile, a major bactericidal component in the SIJ.

## Discussion

A genome survey in the current study revealed the presence of a glycogen metabolism operon in approximately one-third of the *Lactobacillus* species sequenced to date. It was surprising to note that the *glg* operon is absent in several species that are closely related to *L. acidophilus* and are commonly associated with mammalian host environments, notably *Lactobacillus crispatus*, *Lactobacillus gasseri* and *Lactobacillus johnsonii*. Nonetheless, the fact that the majority of the *glg*-encoding species were isolated from mammalian hosts or natural environments, suggests a functional and ecological advantage conferred by the ability to synthesize glycogen. Most *Bifidobacterium* species commonly associated with the human gut environment also possess all the essential enzymes for glycogen biosynthesis and catabolism (Wang and Wise, 2011).

The proposed niche-specific function of glycogen metabolism is also reflected by the inter-strain comparison of *L. helveticus* and *L. bulgaricus*, species that are commonly associated with domesticated dairy environments. The conservation of the genomic region flanking the *glg* operons in *L. helveticus* and *L. acidophilus* led to our conclusion that this operon was lost from the dairy strains of *L. helveticus*. In the case of *L. bulgaricus*, small remnants of the *glg* operon were found in the majority of the sequenced *L. bulgaricus* strains at the genomic region similar to that of the *glg* locus in *L. bulgaricus* PB2003/044-T3-4. This suggested that only the latter strain retained an intact *glg* operon as part of a niche-specific gene repertoire equipped for its adaptation to the human vaginal environment. A recent analysis of 1202 bacterial genomes showed a general correlation between the presence of a complete glycogen pathway with more diverse habitats and flexible lifestyles of bacteria (Wang and Wise, 2011). This supports our observation regarding the preservation of an intact glycogen operon in specific strains of *L. helveticus* and *L. bulgaricus* that have not been adapted to a domesticated environment. Overall, the unique presence of the *glg* operon in specific host-associated strains of *L. helveticus* and *L. bulgaricus*, as well as *L. acidophilus* and other *glg*-encoding *Lactobacillus* species suggests that this operon likely plays an important role in the survival and adaptation of these strains in their niches.

The differential expression of the *glg* operon under various carbohydrate conditions demonstrated that glycogen metabolism in this microorganism was dependent on the type of sugar substrates and availability. The repression effect of glucose observed on gene expression and glycogen accumulation, as well as the presence of a putative *cre* sequence upstream of the *glg* operon, also strongly indicate that the glycogen metabolism is subjected to catabolite repression by glucose. In contrast, glycogen accumulation and *glg* expression were induced in the presence of glucose in microorganisms such as *C. glutamicum*, *S. mutans* and *Salmonella enteritidis* (Spatafora *et al.*, 1995; Bonafonte *et al.*, 2000; Seibold *et al.*, 2007). From the perspective of a specialized niche in the small GI environment, we propose that *L. acidophilus* may sense the presence of glucose, a preferred carbon substrate, as a signal for nutrient abundance and ideal conditions for cell multiplication, which leads the cells to prioritize glucose uptake and carbon flow towards glycolysis and other biosynthetic pathways, promoting rapid growth. Conversely, when glucose or other preferred carbon substrates are absent or exhausted, cells may respond by de-repressing the machinery for the metabolism of complex carbohydrates and activating the glycogen metabolism pathway. Under this condition, glycogen metabolism may synthesize energy storage compounds and serve as a carbon capacitor in order to regulate downstream metabolic flux, presumably in an energy conservation mode for survival maintenance.

Our results demonstrate that the trisaccharide raffinose is among the sugar substrates examined that induced the highest level of the *glg* operon expression and glycogen accumulation. In *L. acidophilus*, raffinose uptake via an ATP-binding cassette (ABC) transporter is hydrolysed into galactose and sucrose (Barrangou *et al.*, 2006; Andersen *et al.*, 2012), and the subsequent catabolism of both galactose and sucrose yields glucose-1-phosphate intermediates that can be directly shunted into the glycogen synthesis pathway, or directed towards glycolysis after conversion to glucose-6-phosphate. The distinct lag phase and high level of glycogen content in raffinose-grown cells throughout growth suggest that the flow of the raffinose catabolite intermediates was likely directed towards glycogen accumulation over glycolysis. In comparison, trehalose is transported via a phosphoenolpyruvate-dependent phosphotransferase system (PTS) and subsequently hydrolysed into glucose and glucose-6-phosphate (Duong *et al.*, 2006) that can be channelled directly into glycolysis, or converted into glucose-1-phosphate by phosphoglucomutase for glycogen synthesis. Thus, it is likely that the uptake and metabolism of trehalose requires less energy (PTS versus ABC transporter) and less complex catabolic enzymatic steps than raffinose, which may explain the slightly slower growth rate on raffinose, and a faster glycogen accumulation rate in trehalose-grown cells during early log phase.

We did not observe a direct correlation between the expression level of the *glg* operon and glycogen accumulation pattern. These observations along with the co-transcription of the *glg* genes provide clues that the glycogen synthesis and catabolism enzymes in *L. acidophilus* may be co-ordinately expressed; a mechanism which was proposed to maintain the glycogen structures and function as a carbon capacitor for sensitive regulation of downstream carbon and energy fluxes (Belanger and Hatfull, 1999; Dauvillee *et al.*, 2005; Seibold and Eikmanns, 2007; Shim *et al.*, 2009). The balance of the parallel synthesis and degradation pathways is in turn regulated by the specificities of the enzymes as well as the state of carbon flux. It is noteworthy that, unlike cells grown on trehalose or glucose (data not shown), the raffinose-grown cells showed remarkably stable intracellular glycogen reserves during prolonged growth periods. Based on these observations, we further speculated that *L. acidophilus* may maintain a higher level of intracellular glycogen to enhance sustainability when growing on a more complex carbon source, a scenario most likely encountered in the GI environment. This proposes a novel strategy for utilizing raffinose and potentially other prebiotic oligosaccharides to enhance the residence time of *L. acidophilus* in the GI environment by inducing glycogen accumulation and co-ordinated regulation of carbon flux for energy of maintenance.

Intriguingly, secondary induction of the *glg* expression was observed during late stationary phase, regardless of the carbon growth substrates, along with a significant increase in expression even in the absence of carbohydrate supplementation. Perhaps this might be partly due to the de-repression of the *glg* operon in the absence of a fermentable carbon source. Another likely reason may be that the *glg* operon was induced during transient starvation conditions, presumably for breakdown of remaining glycogen storage for energy of maintenance, or as part of a stress response regulon. For cells in SDM without any carbohydrate, the inoculum was late stationary-phase cultures grown on trehalose or glucose (see *Experimental procedures*) which were shown to be depleted of intracellular glycogen. In other words, glycogen synthesis or degradation is less likely to occur in glycogen-depleted cells in the absence of carbon source. Hence, the elevated expression of the *glg* operon may serve other physiological functions under carbohydrate-starved conditions.

In the present study, we showed that both *glgA* and *glgB* were essential for the formation of intracellular glycogen. The apparent growth defect of both glycogen synthesis mutants on raffinose also demonstrates that normal growth on raffinose as a sole carbon source relies on the ability of the cells to synthesize intracellular glycogen. In the absence of glycogen synthase or glycogen-branching enzyme, the predicted major influx of glucose-1-phosphate intermediates resulting from raffinose catabolism into the glycogen synthesis pathway may lead to the accumulation of ADP-glucose and linear α-glucan chains in the Δ*glgA* and Δ*glgB* mutants respectively. This may consequently affect carbon down flow and negatively affect growth. In *M. tuberculosis*, GlgB is essential and deletion of *glgB* was unsuccessful (Sambou *et al.*, 2008). It was proposed that the intracellular accumulation of poorly soluble linear α-glucan polymers due to GlgB deficiency was lethal to *Mycobacterium*. The presence of an adenosine diphosphate sugar pyrophosphatase (AspP) in *E. coli* catalyses the breakdown of ADP-glucose and plays a pivotal role in diverting carbon flow from glycogen synthesis to other metabolic pathways in response to physiological status of the cells (Moreno-Bruna *et al.*, 2001). No AspP orthologue was identified in the genome of *L. acidophilus*. It is unclear whether *L. acidophilus* encodes an alternative mechanism that may sense ADP-glucose accumulation and divert carbon flow from glycogen anabolism.

Both Δ*glgB* and Δ*glgP* mutants were more susceptible to the exposure of bile and simulated SIJ. It remains to be determined whether the bile sensitivity phenotype exhibited by both mutants was due to an overall growth defect compromising the functionality of the bile efflux systems (Pfeiler and Klaenhammer, 2009), and other resistance mechanisms that protect the cells from digestive enzymes (i.e. amylase, protease, lipase) present in the SIJ. Given that the same phenotype was not observed in the other glycogen-deficient mutant, Δ*glgA*, we speculate that in the Δ*glgB* mutant, the predicted accumulation of the α-glucan polymers that were unable to form glycogen structures might result in various physiological perturbations that affected its growth and potentially survival in the small intestinal environment. It was previously shown in *M. smegmatis* that the mutation of the GlgE glycogen-degrading enzyme resulted in slower growth and morphological alteration (Belanger and Hatfull, 1999). Based on the model of glycogen metabolism as a carbon capacitor, the absence of GlgE disrupts glycogen recycling, resulting in glucose molecules sequestered in glycogen, thereby compromising downstream metabolic pathways. Likewise, the growth and bile sensitivity phenotypes of the *L. acidophilus* Δ*glgP* mutant emphasize the physiological importance of co-ordinated glycogen synthesis and degradation as well as the ability of the cells to retrieve a carbon source from glycogen storage during normal growth and stress conditions.

Overall, this study provides insights into the physiological importance of glycogen metabolism in *L. acidophilus*, which will also likely be highly relevant in other *Lactobacillus* species. We hypothesized that the concerted glycogen anabolism and catabolism in *L. acidophilus* serve important roles in maintaining the intricate balance of the carbon central metabolism network, operating based on nutrient status, and influencing various physiological functions and probiotic attributes of the microbe. Further studies are warranted to establish and exploit the biological role of glycogen metabolism *in vivo*, in order to optimize the biodelivery and probiotic activities of *L. acidophilus* in the host environment.

## Experimental procedures

### Bacterial strains and growth conditions

The bacterial strains and plasmids used in this study are summarized in Table 1. *L. acidophilus* strains were propagated in MRS broth (Difco Laboratories, Detroit, MI) statically under aerobic conditions or on MRS agar [1.5% (w/v), Difco] under anaerobic conditions at 37°C or 42°C, where indicated. Recombinant strains were selected in the presence of 2 μg ml^−1^ of erythromycin (Sigma-Aldrich, St. Louis, MO) and/or 2–5 μg ml^−1^ of chloramphenicol (Sigma) when appropriate. For *upp*-based counterselectable gene replacement procedures (Goh *et al.*, 2009), plasmid-free double recombinants (of which a double-crossover event had occurred whereby the integrated plasmid backbone was excised from the chromosome) were selected on a semi-defined agar medium containing 2% (w/v) glucose (GSDM) (Kimmel and Roberts, 1998) and 100 μg ml^−1^ of 5-fluorouracil (5-FU) (Sigma) as described previously (Goh *et al.*, 2009). For gene expression and glycogen accumulation studies, cells were grown in semi-defined medium (Kimmel and Roberts, 1998) (SDM) with glucose substituted for the carbohydrates examined. For growth experiments, overnight cultures were inoculated at 1% (v/v) into 96-well microplate wells (Corning Costar, Corning, NY) in triplicate, each containing 200 μl of SDM supplemented with 1% of the carbohydrate. Microplates were sealed with clear Thermalseal film (ISC Bioexpress, Kaysville, UT), incubated at 37°C in a Fluostar Optima microplate reader (BMG Labtech, Cary, NC) and optical density of cells was monitored at 600 nm (OD_600_) for 48 h.

*Escherichia coli* strains were grown in Brain Heart Infusion (BHI) (Difco) medium at 37°C with aeration. *E. coli* EC101 cloning host (Law *et al.*, 1995) was propagated in the presence of 40 μg ml^−1^ of kanamycin. When necessary, erythromycin was added at a final concentration of 150 μg ml^−1^.

### DNA manipulations and transformation

Standard DNA techniques, genomic and plasmid DNA isolations, and transformations were performed as described previously (Goh *et al.*, 2009). PCR primers (Table S1) were synthesized by Integrated DNA Technologies (Coralville, IA). For cloning and DNA sequencing, PCR amplicons were generated using PfuUltra II Fusion HS DNA polymerase (Agilent, Santa Clara, CA) based on manufacturer's instructions.

### Sequence analysis

Rho-independent transcriptional terminators were predicted by TransTermHP (Kingsford *et al.*, 2007). Deduced protein sequences were compared against the non-redundant protein database using BlastP (http://blast.ncbi.nlm.nih.gov/Blast.cgi). The protein sequence of the characterized GlgA from *C. glutamicum* (Tzvetkov *et al.*, 2003) was used to search for GlgA orthologues encoded in the bifidobacterial genomes. Multiple sequence alignments of GlgA orthologues were carried out in clustalx v2.1 (Larkin *et al.*, 2007) with BLOSUM series protein weight matrix and clustering was performed with neighbour-joining method. Unrooted phylogenetic tree generated from clustalx v2.1 was visualized using MEGA 5.1 software package (Tamura *et al.*, 2011).

### RT-qPCR and RT-PCR assays

For RT-qPCR analysis of the *glg* operon expression during growth on glucose, trehalose, lactose and raffinose, *L. acidophilus* strain NCK1909 was subcultured once in SDM containing 1% (w/v) of each sugar for 24 h. These cultures were used to inoculate at 2% (v/v) into 50 ml of SDM with 2% of each respective sugar. The cultures were grown to mid-log phase (OD_600_ ∼ 0.6) and harvested by centrifugation at 1717 *g* for 5 min at room temperature. Two control cultures were also included where NCK1909 subcultured in SDM with 1% glucose or trehalose above was used to inoculate (2%) 50 ml of SDM without carbohydrate. These control cultures, which only reached a maximal final OD_600_ of 0.1–0.2 in the absence of carbohydrate, were harvested along with the mid-log phase cultures grown on raffinose (*c*. 10 h of growth). Harvested cell pellets were flash-frozen in an ethanol-dry ice bath and stored at −80°C. Samples were collected from two independent experimental replicates (representing two biological replicates). Total RNA was extracted, DNase-treated and purified as described previously (Goh *et al.*, 2006) for RT-qPCR assays with the *glgA* gene as target for PCR amplification. The absence of genomic DNA in purified RNA samples was verified by PCR using NCFM gene-specific primers. The iScript One-step RT-PCR kit with SYBR Green (Bio-Rad Laboratories, Hercules, CA) was used for RT-qPCR according to manufacturer's suggestions, with each reaction scaled down to a total volume of 25 μl containing 50 ng of RNA template and 300 nM final concentration of each primer glgArt.F/glgArt.R (Table S1). RT-qPCR was performed with an iCycler MyiQ single colour detection system (Bio-Rad). Data were analysed using iCycler MyiQ software v1.0 (Bio-Rad). The number of threshold cycles per well were determined using the auto calculated ‘threshold cycle calculation’ and ‘PCR base line subtracted curve fit’ analysis mode. Transcript copy numbers of *glgA* were quantified from the standard curve generated from known concentrations of the *glgA* PCR product.

To determine whether the *glg-amy-pgm* genes are co-transcribed as a single mRNA with RT-PCR assay, DNase-treated and purified RNA samples from cultures grown on raffinose, or without carbohydrate supplementation, were reverse-transcribed to generate first-strand cDNA using a Superscript III First-Strand Synthesis System (Life Technologies, Grand Island, NY) according to manufacturer's recommendation, with 5 μg of RNA and 50 ng of random hexamers. A negative control reaction was also included where no reverse transcriptase was added. Following hydrolysis of RNA template with *E. coli* RNase H, aliquots (2 μl) of the RT reactions were used as PCR amplification templates in standard 50 μl PCR reactions containing each of the primer pairs (Table S1) that spanned throughout the entire gene cluster.

For temporal *glg* operon expression analysis, NCK1909 was pre-cultivated in SDM containing 2% (w/v) raffinose or trehalose and then inoculated at 2% (v/v) into 600 ml of SDM containing 2% of each respective sugar. Growth was monitored by OD_600_ and aliquots of cells were harvested at various growth phases [early log (OD_600_ ∼ 0.3), mid-log (OD_600_ ∼ 0.6), early stationary (OD_600_ ∼ 1.0), stationary (OD_600_ ∼ 1.5) and late stationary (OD_600_ ∼ 2.2)] for RT-qPCR analysis and intracellular glycogen measurement. Samples were collected from two independent biological replicates. RNA purification and RT-qPCR analysis were performed as described above.

### Intracellular glycogen assays

For qualitative detection of intracellular glycogen in *L. acidophilus*, procedures for iodine staining of colonies were adapted from previous reports (Govons *et al.*, 1969; Law *et al.*, 1995). Briefly, 5–10 μl of overnight cultures were spotted on SDM agar medium containing 2% (w/v) trehalose, allowed to absorbed and incubated at 37°C anaerobically for 48 h. The colonies were flooded with freshly prepared iodine solution (0.01 M I_2_, 0.03 M KI; 5 ml per plate), incubated in the dark for 1 min, and the iodine solution was removed with a pipet. Cells containing intracellular glycogen stained brown, whereas glycogen-deficient cells appeared as yellow or colourless.

For quantitative measurement of glycogen content, glycogen extraction was performed based on alkaline digestion at 95°C (Parrou and Francois, 1997). For this purpose, aliquots of cells were harvested at indicated growth phases by centrifugation and stored at −80°C for further analysis. Each cell pellet was thawed and resuspended in 1 ml of phosphate-buffered saline (PBS, pH 7.4; Life Technologies), transferred to sterile pre-weighed 1.5 ml screw-capped tube and centrifuged (14 549 *g* for 1 min) to remove all culture supernatant. Centrifugation was repeated at least twice to remove all culture supernatant in order to obtain an accurate cell wet weight. The tubes with the cell pellets were weighed once again (to obtain net cell wet weight) before resuspending each cell pellet in 0.25 ml of 0.25 M Na_2_CO_3_. The cell suspensions were incubated at 95°C for 4 h and brought to pH 5.2 by adding 0.15 ml of 1 M acetic acid and 0.6 ml of 0.2 M sodium acetate (pH 5.2). Then, amyloglucosidase (Roche Diagnostics, Indianapolis, IN) was added to the cell suspensions at 1.4 U ml^−1^ final concentration and incubated at 57°C overnight with constant agitation in a hybridization oven. The cell suspensions were centrifuged at 5000 *g* for 3 min, and the glucose released was determined using a hexokinase/glucose-6-phosphate dehydrogenase-based glucose assay kit (Sigma) per manufacturer's instructions. Intracellular glycogen content was expressed as mg of glucose (released from glycogen by amyloglucosidase) per g of cell wet weight.

### Construction of deletion mutants

The *glgA*, *glgB*, *glgP* and *amy* genes from *L. acidophilus* NCFM were deleted using a *upp*-based counterselectable gene replacement system (Goh *et al.*, 2009). For each deletion, the in-frame deletion was constructed by first amplifying DNA segments flanking the regions upstream and downstream of the deletion target: (i) for *glgA* deletion, a 1347 bp deletion (94% of gene) was constructed by PCR of 775 bp and a 788 bp DNA segments respectively using glgA1/glgA2 and glgA3/glgA4 primer pairs (Table S1), (ii) for *glgB* deletion, a 1779 bp deletion (93% of gene) was constructed by PCR of 655 bp and a 636 bp DNA segments respectively using glgB1/glgB2 and glgB3/glgB4, (iii) for *glgP* deletion, a 2280 bp deletion (95% of gene) was constructed by PCR of 700 bp and 619 bp DNA segments respectively using glgP1/glgP2 and glgP3/glgP4, and (iv) for *amy* deletion, a 1674 bp deletion (95% of gene) was constructed by PCR of 640 bp and 629 bp DNA segments respectively using amy1/amy2 and amy3/amy4. Next, purified PCR products of both DNA segments corresponding to each target were fused and amplified to generate copies of deletion alleles via splicing by overlap extension PCR (SOE-PCR) (Horton *et al.*, 1989), using 10 ng of each PCR product as amplification templates in a 50 μl PCR reaction with primer pair glgA1/glgA4, glgB1/glgB4, glgP1/glgP4 or amy1/amy4 respectively. All PCRs were performed with 25–30 amplification cycles. Construction of recombinant integration plasmids pTRK1042, 1043, 1071 and 1072 (Table 1) and the recovery of double recombinants were performed as described previously (Goh *et al.*, 2009; Goh and Klaenhammer, 2010). Double recombinants carrying Δ*glgA*, Δ*glgB*, Δ*glgP* or Δ*amy* allele were screened by colony PCR using primer pair glgA5/glgA6, glgB5/glgB6, glgP5/glgP6 or amy5/amy6 (Table S1), respectively, which specifically anneal to the flanking regions of the deletion targets. In-frame deletions and sequence integrity were confirmed by DNA sequencing using the above primer pairs.

### Exposure to simulated gastric and small intestinal juices

Challenge assays were performed essentially as described previously (Charteris *et al.*, 1998; Frece *et al.*, 2005; Goh and Klaenhammer, 2010). Briefly, overnight cultures were pelleted, washed twice and resuspended in sterile distilled water. The cell suspension (0.2 ml) was added to 1 ml of freshly prepared simulated gastric juice [SGJ; 0.5% (w/v) NaCl solution containing 3 g l^−1^ pepsin (Fisher Scientific, Pittsburg, PA), pH 2.0] or small intestinal juice [SIJ; 0.5% NaCl solution containing 1 g l^−1^ pancreatin (Sigma) and 3 g l^−1^ Oxgall (Difco), pH 8.0] and incubated at 37°C. Viable cell count was determined by plating onto MRS agar at 30 min or 1 h intervals.
