# Supplementary material for: A functional glycogen biosynthesis pathway in Lactobacillus acidophilus: expression and analysis of the glg operon
Source: Mol Microbiol. 2013 Aug 16;89(6):1187–200. doi: 10.1111/mmi.12338 (PMC4282360; doi:10.1111/mmi.12338)
Supplement: Supplementary file 1 — Supporting Information [file mmi0089-1187-SD1.pdf]

Supplemental figure legends (Goh and Klaenhammer)

Fig. S1. (A) Genetic organization of the *L. acidophilus* NCFM glycogen metabolism gene cluster. Solid horizontal lines indicate the primer pair targets (1 to 5) for RT-PCR analysis. (B) RT-PCR results showing PCR-amplified products corresponding to the primer pair targets in (A). W, positive control using wild-type NCFM genomic DNA as PCR template; +RT, cDNA synthesis reaction with added Superscript III reverse transcriptase as PCR template; -RT, negative control, cDNA synthesis reaction without reverse transcriptase as PCR template.

Fig. S2. Growth of *L. acidophilus* glycogen metabolism mutants compared to the parent strain (NCK1909) in MRS medium as measured by OD_{600} (A) and plating and enumeration of viable cells on MRS solid medium (B). Both Δ*glgB* and Δ*glgP* mutants exhibited slightly slower growth compared to the parent and other mutant strains.

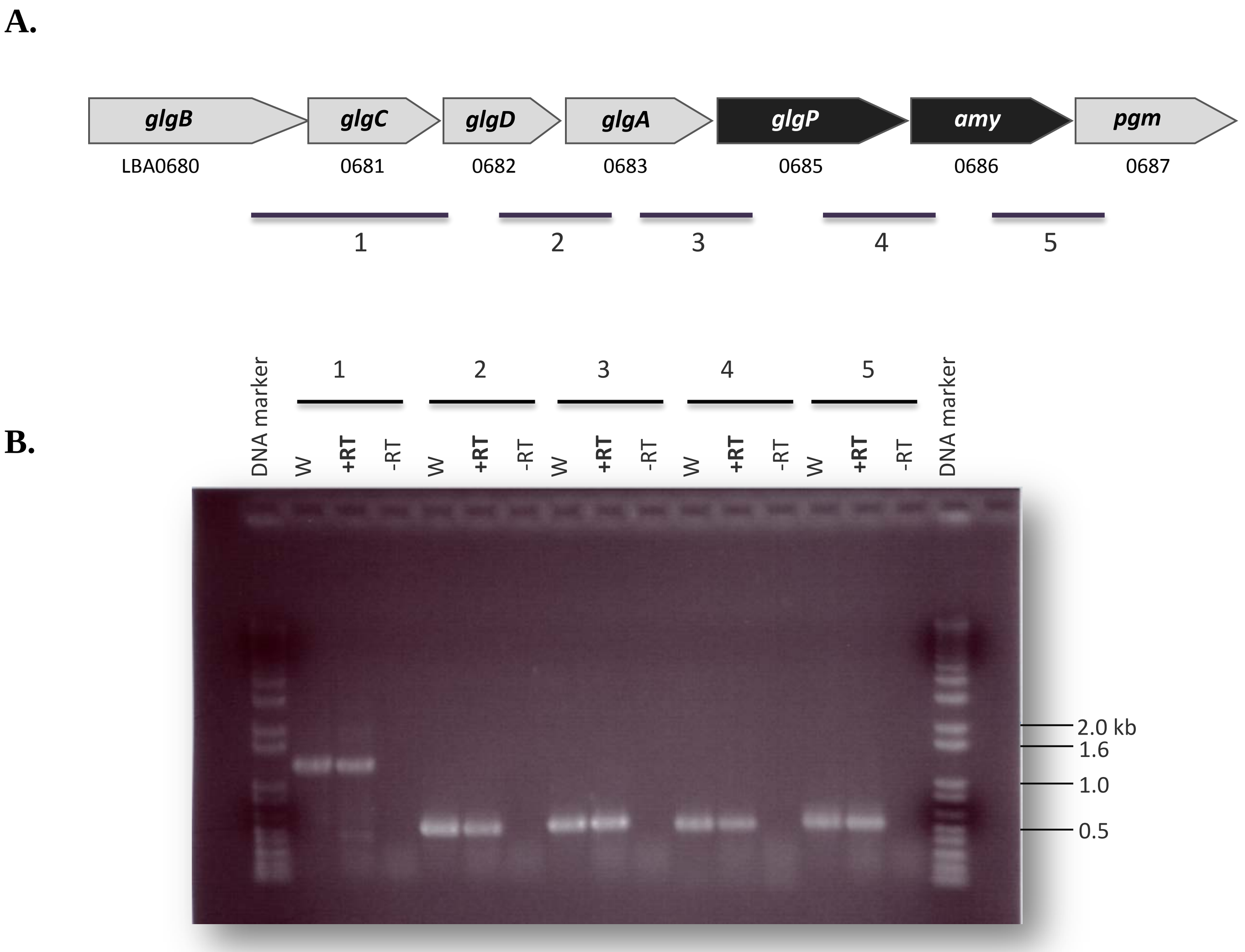

Fig. S1. Goh and Klaenhammer

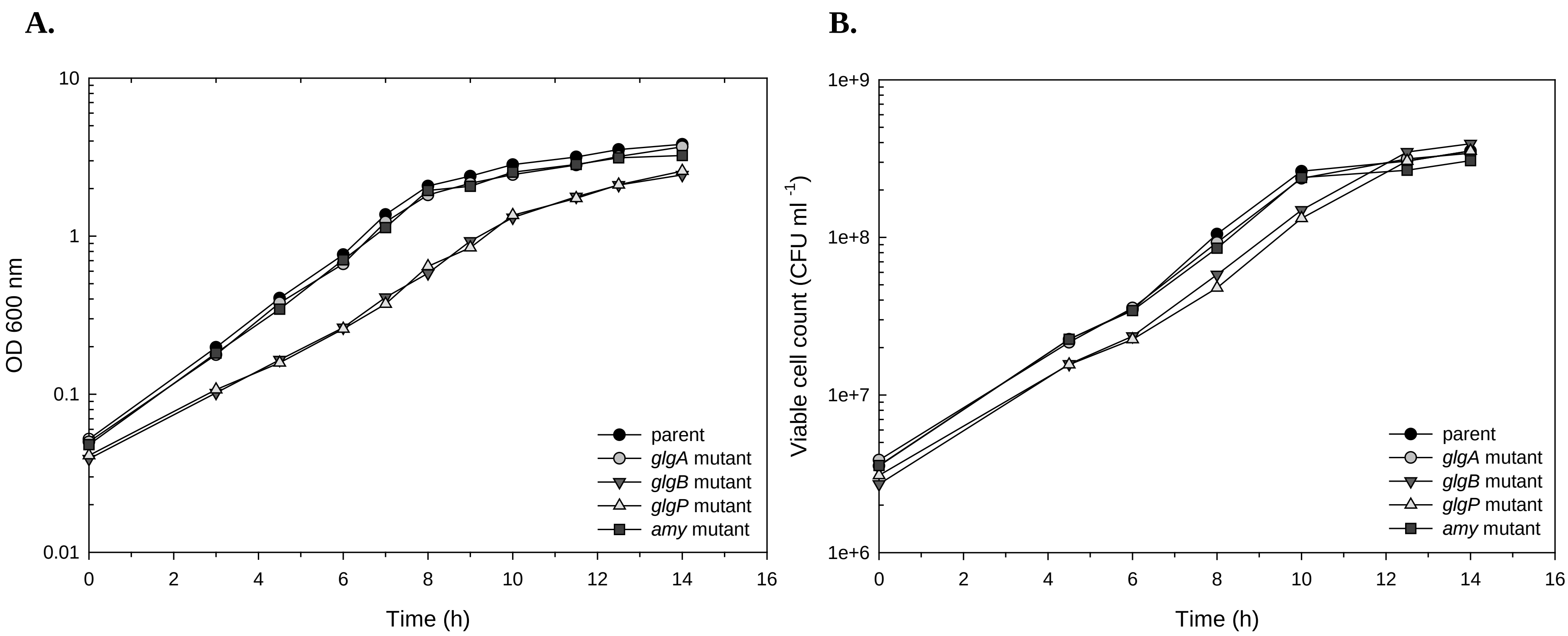

Fig. S2. Goh and Klaenhammer

Table S1. Oligonucleotide primers used in this study (Goh and Klaenhammer)

Primers	Sequence (5' to 3')[a]
RT-qPCR analysis	
glgArt.F	GAT AGT GGT ATT GTC CGA ATG G
glgArt.R	AAT CGC ATC TAA GCC TTG TC
RT-PCR analysis	
(1) glgBCD.F	CCG GGT TTT AGT GCT GTG AT
(1) glgBCD.R	TGC GAT ATT TAC CTG CGA AA
(2) glgD-A.F	GCA ACT GGC TGT AGA ATA AAT GG
(2) glgD-A.R	CAT AAA TTC CCG GAC GCT TA
(3) glgA-P.F	CTG GCT TTG GCT TTA AGG AA
(3) glgA-P.R	ATA AAA GCT GCA GCC AAT CG
(4) glgP-amy.F	TGG GCT CAA ATG AGT TTG GT
(4) glgP-amy.R	TTC CAT TAG CAA AGC GAT CC
(5) amy-pgm.F	TTG TCG GCT GGG GTT AAT AG
(5) amy-pgm.R	CAT CAA TCA AAC GCG CTA AA
Construction, screening, and sequence verification of gene deletions	
glgA1	GTA ATA GGA TTC CCA TCA AGA AAA TGG AAA TCG
glgA2	AAA AGG TGC ACA TTC AGC AC
glgA3	GTG CTG AAT GTG CAC CTT TTT CTG CTG ATA AGT ATA AGT GGA TG
glgA4	TAA AGT AGA GCT C GG ATA TGA CAA TTC TTC TTC TGG TG
glgA5	TTA TTG CAA AGC CAA GCA AA
glgA6	TGA ACA GCC ACG TAA TCT GG
glgB1	GAT TAT AAG CTT CCG TCG TGA TCA ATT ACG TG
glgB2	TTG AAG ATA TAG CTC ATT GCC
glgB3	GCA ATG AGC TAT ATC TTC AAG CTG TGA TTT TAA AAC CTG TAG ATG
glgB4	TTA GTA GAA TTC TGG ATG CTC AGG CTT TTC TT
glgB5	AAT GCG GTT GGT GAA GGT AG
glgB6	CGT GCC CTC AAA CCA TTT AC
glgP1	GTA ATA GGA TCC GGA TAA AAT AGC ACT TCA AAA AG
glgP2	TTC ATC AAG TTC TTC TTC AAA A
glgP3	TTG AAG AAG AAC TTG ATG AAG CAC AAG ATA TTT GGC ATT T
glgP4	TAA AGT AGA GCT C TT TCT AAT TCC AGT TAA ATT ACC G
glgP5	TCC CAA GAC TGA TCC CGT AA
glgP6	TGG ATT AAG ATA AAG GAC TGT AAC ACC
amy1	GTA ATA GGA TCC ACG GCA AGT TAA AGG TAA TC
amy2	CTG CTC TTG CCA AGA ATC AT
amy3	ATG ATT CTT GGC AAG AGC AGG ATA CTT TGG ACC AAA TTC AAC
amy4	TAA AGT AGA GCT C TA GCA TTA TGC GAA GCA GTG
amy5	AGG GAT TGA TCA TCC TAA GC
amy6	ACT GAA GCA ACT TCA ACA CC

[a] restriction enzyme sites, underlined
